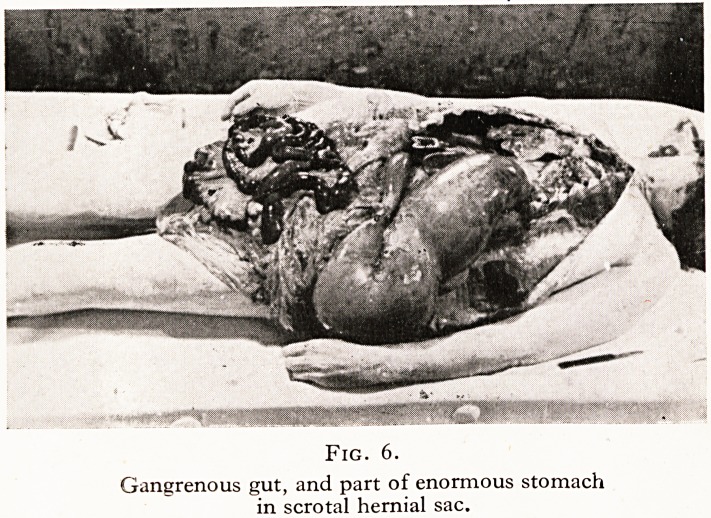# Some Post-Mortem Surprises

**Published:** 1950-01

**Authors:** F. D. M. Hocking

**Affiliations:** Pathologist to the Royal Cornwall Infirmary, Truro


### SOME POST-MORTEM SURPRISES

*The Presidential Address*
*to the Cornwall Clinical Society, June 6th, 1949*

BY

F. D. M. HOCKING, M.SC., M.B., F.R.I.C.
*Pathologist to the Royal Cornwall Infirmary, Truro.*

---

All practitioners must be aware how very different is the hold on life of individuals. Some persons are able to withstand terrific pathological punishment before they succumb. For example, a man sixty-six years of age dropped dead in the street; no medical history was available and no doctor had attended him for many years, yet post-mortem examination showed an enormous liver weighing nearly 110 ounces in which apparently the whole of the normal tissue had been replaced by a primary carcinoma. A somewhat similar case was that of a woman, fifty-one years of age, who also dropped dead in the street; she had no medical history, and yet at the post-mortem examination we found an abdomen apparently solid with an ovarian carcinoma, in which the coils of both large and small gut were completely embedded and encased. (Figure 1.)

Most doctors are, of course, familiar with the man with " cor bovinum " who lives, perhaps, to a very old age, with some disability but by no means totally incapacitated. But at the other end of the scale there is the man, usually young, who develops a small patch of acute atheroma in a coronary artery and who is dead within a few months, because there has been no time for collateral circulation to re-establish the nourishment of the heart muscle: all that is found at the post-mortem examination is a coronary artery almost completely occluded, generally a quarter of an inch from its origin and for a distance of one-quarter to one-half an inch.

Amongst the " trivial " causes of sudden death, vagal shock is undoubtedly the most important; this shock can arise from stimulation of any of the terminations of the parasympathetic nervous system—a rather frightening thought for the modern practitioner who is encouraged to use needles and other instruments on his patient with increasing frequency. Fortunately, sudden death from such an accident is rare; unfortunately, it is quite impossible to predict when it is likely to happen. It may even happen under surgical anaesthesia. I have seen two cases this year, both within a week, where the withdrawal of an intratracheal tube had caused sudden death although the patient was still anaesthetized. Light pressure on the larynx may also cause a vagal shock death. Sometimes this pressure is so light as to cause insignificant and apparently quite trivial bruising, and on occasion, there may be found no bruising at all—and nothing whatever to discover as a cause of death. It is vitally important in these cases to have an absolutely bloodless dissection of the neck. This, in my opinion, can be achieved only by dissecting the neck at the very end of the post-mortem examination. The technique adopted is to examine the abdomen first, and before opening the chest; concluding by cutting up the abdominal aorta and vena cava, thus drawing most of the blood into the abdominal cavity from which it can be swabbed out. The chest is examined by cutting the ribs through in the usual way but only up to the level of the second rib, the sternum being removed by shearing through the sterno-manubrial junction. In this way the great veins of the neck are not disturbed and the thoracic viscera are examined quite dry before their removal. In the case of young women who may have been aborted the procedure is, of course, reversed: the thoracic cavity is opened as above before disturbing the abdominal viscera, and the heart and blood vessels are first of all examined for the presence of air—or in the case of an apparently trivial fracture causing unexpected death, for fat. After the thoracic viscera are removed, the brain is taken out and then any remaining blood in the neck is drained by gravity into the thoracic cavity and mopped out. Only then is it safe to undertake the dissection of the neck. The field by this time is quite bloodless so that no possible confusion can exist as to whether a bruise is an ante-mortem bruise or a dissection artefact. All suspicious areas are, of course, examined microscopically, but if these precautions are taken some awkward questions during a cross-examination can be dealt with confidently.

There is still some controversy about the part played by " Status Lymphaticus " in association with death from trivial causes. Experience during the years since the Pathological Society's Report on this subject tends to confirm the findings of the committee of investigation. All cases that might temptingly be ascribed to *status lymphaticus* can be covered, and covered more satisfactorily, under the heading of vagal shock. They occur practically always in children on whom throat operations are performed, or who have been found dead in their cots or perambulators. There can be little doubt that the former are cases of vagal shock. The latter are more difficult to assess. Many show acute respiratory infections, mainly acute bronchiolitis which can kill an infant in a matter of hours, with or without signs of suffocation. The only really satisfactory course to adopt in these cases is to withhold an opinion until after microscopical examination of the lungs, and not to allow oneself to be rushed by the Coroner or Police.

Then there are the cases in which the apparently obvious cause of death is not the actual cause. For example, a man known to be suffering from advanced disease of the heart was seen to be running for a bus when he stumbled and fell backwards. At the post-mortem examination, he showed an advanced degree of aortic calcification, coronary disease and cardiac hypertrophy that might be expected to be associated with sudden heart failure due to undue exertion. But he happened to be insured for a large sum of money against death by accident, so that a complete post-mortem examination was made. It was discovered that the dead man had suffered a subluxation of the third and fourth cervical vertebrae so that the spinal cord was nipped and bruised. (Figure 2.) Death had been due actually to shock associated with the injury, and not to the rather more obvious cardiac lesion: the widow duly collected the insurance money.

On another occasion, a girl was found dead one morning in a tent in a Youth Hostel camp. She showed all the classical signs of death by suffocation; spermatozoa were found in the vagina and very strong suspicion rested upon a certain member of the community whose obvious attentions were equally obviously unwelcome. The young man vehemently protested his innocence and said also that he thought the girl was behaving somewhat peculiarly the night before her death. On account of this numerous sections were taken for microscopical examination. The spinal cord, pons, medulla and bulb all showed an intense perivascual cuffing with lymphocytes and there was no doubt that the girl had died of acute bulbar paralysis due to polio-encephalitis! (Figure 3.)

An elderly woman was found dead in bed, and post-mortem examination showed a large infarct of the anterior wall of the left ventricle some two inches in diameter, quite sufficient to explain her death. The appearance of the lungs, however, was not consistent with this: they were intensely engorged and showed the plum-purple colour so often associated with influenzal pneumonia or poisoning with soporific drugs, particularly the barbiturates. Cerebro-spinal fluid, liver, stomach, kidneys and urine were therefore taken and analysed for poisons: some sixty grains of a barbiturate were recovered. There is no doubt that this woman died of poisoning, the infarct being probably associated and the terminal event.

Abdominal catastrophe may closely simulate irritant poisoning. A woman was taken violently sick after a meal prepared by her husband with whom relations were distinctly strained. She took to her bed and continued vomiting intermittently for four days. It was noted by her daughter that she always vomited most after having been given food and drink by her husband. She also had diarrhoea, and towards the end of the second day of her illness she passed blood-stained stools. She died after four days' illness, no doctor having been called, and a strong suspicion was aroused of arsenical or similar poisoning. The post-mortem examination revealed thrombosis of the superior mesenteric artery. (Figure 4.)

On another occasion, an unmarried woman was admitted to hospital with hyperpyrexia and covered with a haemorrhagic rash very suggestive of thrombocytopaenic purpura. She died before any investigations could be made. At the post-mortem examination the haemorrhagic rash was found to involve all the viscera, including the suprarenals, so that a diagnosis of meningococcal septicaemia or thrombocytopaenic purpura appeared to

### PLATE I

FIG. 1.
Massive ovarian carcinomatosis without apparent symptoms.

FIG. 2.
Bruise of cervical spinal cord due to fall on buttocks.

### PLATE II

Fig. 3.
Spinal cord, cervical region, shewing perivascular cuffing in poliomyelitis.

Fig. 4.
Thrombosis of superior mesenteric artery without gangrene of gut

PLATE III

Fig. 5.
Scrotal hernia of unusual size.

Fig. 6.
Gangrenous gut, and part of enormous stomach in scrotal hernial sac.

be justified. But she was two and a half months pregnant. Could this be a case of poisoning? Examination of the viscera actually extracted some eighty grains of aspirin: this was, in fact, a case of suicide by poisoning.

A most remarkable case was that of a taxi driver who died two days after a minor car accident in which he received a blow of moderate severity in the upper abdomen, by being thrown against the steering wheel. He complained of nothing for twenty-four hours, after which he was seized with vomiting and died within twelve hours without seeing a doctor. At the post-mortem examination we found a scrotal hernia twelve inches in diameter, containing the whole of the gut, large and small, and one-third of a stomach measuring four feet in length and two feet three inches in circumference. The pancreas was drawn over the posterior abdominal wall in the form of a sheet of tissue. The gut was gangrenous. The blow on the abdomen had forced stomach contents and gas into the scrotal portion of the enormous stomach and the resulting pressure had caused strangulation of the gut in the sac. (Figures 5, 6.)

## Figures and Tables

**Fig. 1. f1:**
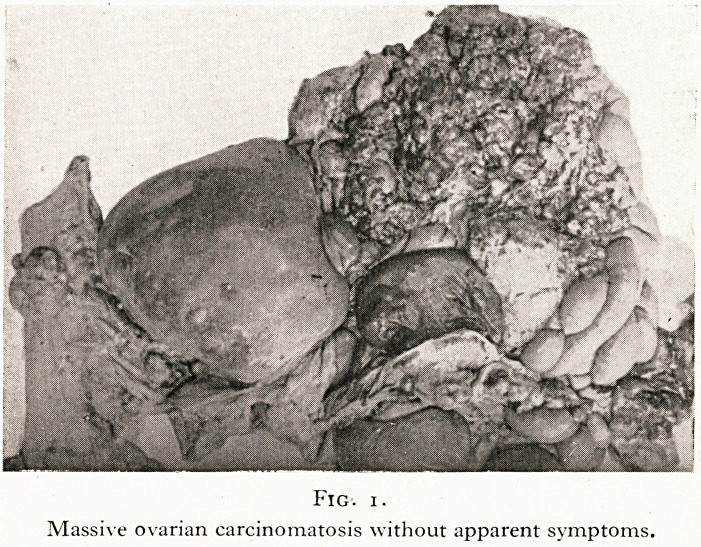


**Fig. 2. f2:**
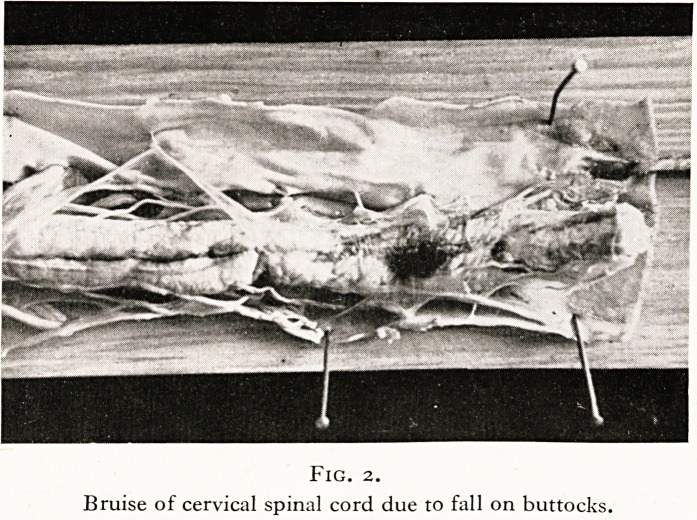


**Fig. 3. f3:**
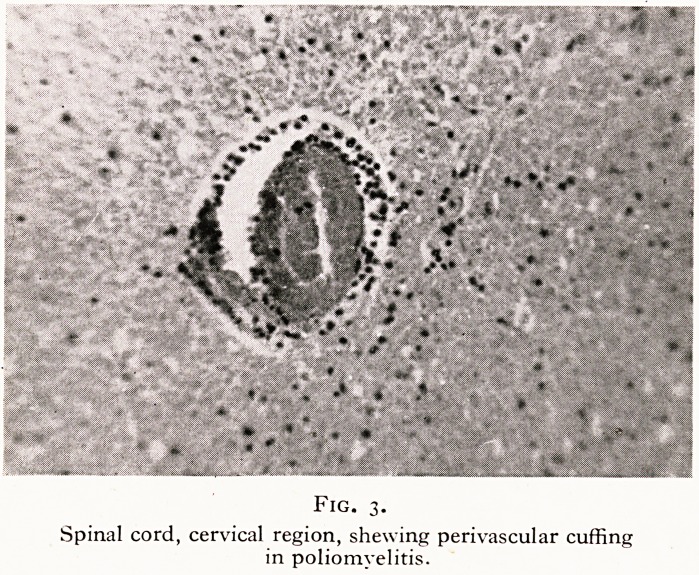


**Fig. 4. f4:**
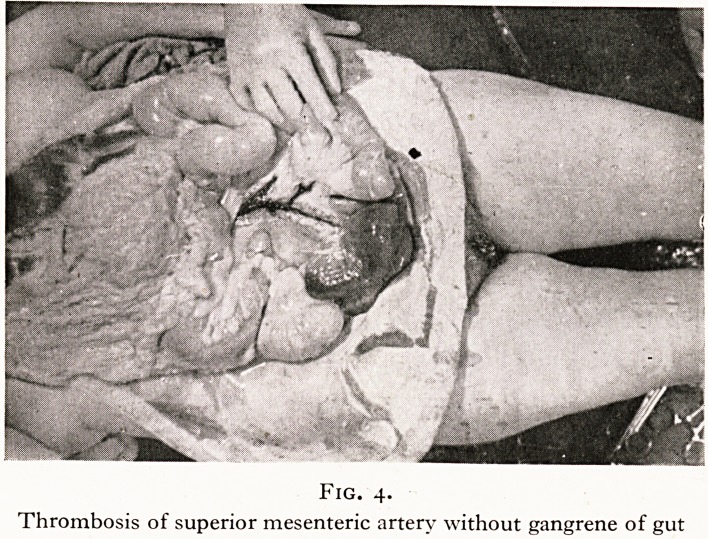


**Fig. 5. f5:**
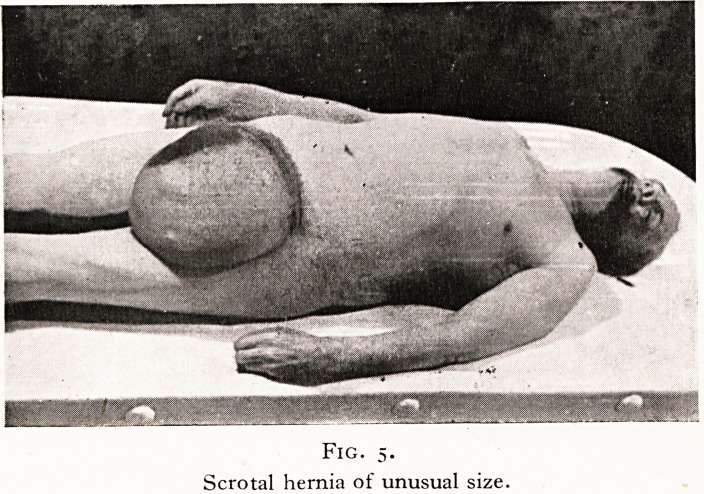


**Fig. 6. f6:**